# Kinematic changes following concurrent epidural injection with autologous conditioned serum (ACS) and glucocorticoids in dogs with cauda equina syndrome

**DOI:** 10.1016/j.vas.2026.100911

**Published:** 2026-10-20

**Authors:** Patrick Blaettler Monnier, Indre Matsuzaki, Nico Gruetter, Julien Troillet

**Affiliations:** a orthoVET, Fasanenstrasse 13, Frenkendorf, CH-4402, Switzerland; b Cavia, Iwamoto 537132, Fuji, Shizuoka, 416-0901, Japan; c FHNW University of Applied Sciences Northwestern Switzerland - Campus Muttenz, Hofackerstrasse 30, 4132 Muttenz, Basel-Land, Switzerland; d Orthogen Veterinary GmbH, Ernst-Schneider-Platz 1, Düsseldorf, 40212, Germany

**Keywords:** Cauda equina syndrome, Chronic pain, Autologous Conditioned Serum (Orthogen ACS), Epidural infiltration, Inertial measurement unit (IMU), Kinematic analysis

## Abstract

Degenerative lumbosacral stenosis (DLSS) causes progressive nerve root compression, leading to cauda equina syndrome (CES) and painful gait impairments in dogs. Epidural co-administration of autologous conditioned serum (ACS) and glucocorticoids (GC) is used as a conservative intervention, but objective outcome data remain lacking. This retrospective exploratory study evaluated whether an inertial measurement unit (IMU)-based kinematic system (LupoGait) could detect gait changes following a single epidural injection of ACS and triamcinolone acetonide in 44 dogs with CES. Nominal post-treatment changes were observed in several hindlimb parameters, including stance phase (HL +0.03, 95% CI 0.005–0.05, *p* = 0.018), maximum gyroscope amplitude (HL +0.32, 95% CI 0.02 to 0.69, *p* = 0.032), and variance of the differentiated gyroscope signal (HL +0.10, 95% CI 0.01 to 0.23, *p* = 0.036). However, no evaluated outcomes remained statistically significant after correction for multiple comparisons. Given the uncontrolled retrospective design, these exploratory findings cannot establish therapeutic efficacy or isolate ACS-specific effects from GC administration. Nevertheless, this study demonstrates the feasibility of IMU-based kinematic analysis for objective longitudinal monitoring in dogs with CES and provides preliminary baseline data for prospective, controlled clinical trials.

## 1 Introduction

Cauda equina syndrome (CES) is a spinal condition caused by prolonged compression of lumbosacral nerve roots. Although rare in humans, it commonly affects dogs and other animal species (Cariou et al., 2008; Melvin et al., 2023; Spector et al., 2008; Šulla et al., 2018). Lumbosacral transitional vertebra (LTV), a congenital anomaly, predisposes dogs to degenerative lumbosacral stenosis (DLSS), which subsequently leads to CES (Berg et al., 2025). This pathological progression occurs frequently in medium and large dogs, such as German Shepherds (Flückiger et al., 2006; Morgan et al., 1993; Suwankong et al., 2008; Worth et al., 2019). Dogs with LTV are eight times more likely to develop clinical signs of CES (Flückiger et al., 2006), and CES symptoms occur in most dogs with DLSS (Suwankong et al., 2008).

Progressive cauda equina compression affects nerve-root blood flow and axonal function, causing inflammation and worsening neurological deficits (Kobayashi et al., 2004). Early clinical signs include lumbar pain and lameness, which can progress to paresis and paralysis (Gernone et al., 2022; Medina-Serra et al., 2025; Suwankong et al., 2008; Šulla et al., 2018). Diagnosis relies on clinical symptoms, orthopedic evaluation, and multimodal imaging (Morgan, 1999; Suwankong et al., 2008).

Treatment options range from conservative management with systemic nonsteroidal anti-inflammatory drugs (NSAIDs) or corticosteroids to surgical decompression. Although surgical intervention shows a high success rate (78%), it carries complication risks (De Decker et al., 2014; De Risio et al., 2001; Šulla et al., 2018; Worth et al., 2019). Currently, no therapeutic consensus exists due to a lack of direct comparison studies, and all options carry potential adverse effects (Goh, 2023; Šulla et al., 2018). Consequently, interest in local, minimally invasive techniques in veterinary medicine has grown. Epidural corticosteroid injections can relieve clinical signs, particularly in early stages or non-surgical candidates. For example, a single epidural injection of methylprednisolone in dogs with DLSS provided long-term clinical improvement in 15.6% of patients, though some relapsed after two months (Gomes et al., 2020).

In addition to corticosteroids, orthobiologic therapies have gained attention to modulate immune signaling and support endogenous repair, rather than broadly suppressing inflammation (Costa et al., 2025; Wernecke et al., 2015). Autologous conditioned serum (ACS) – an acellular blood derivative produced via extended whole-blood coagulation, is an established orthobiologic in human and veterinary musculoskeletal medicine (Evans et al., 2016; Frisbie et al., 2007; Geburek et al., 2015; Hang et al., 2024; Wehling et al., 2007). In dogs, concurrent intra-articular administration of ACS and triamcinolone acetonide resulted in superior long-term clinical improvements compared with injection of the individual components (Alves et al., 2022a, 2022b). In human patients, intrathecal or epidural ACS injections are used to manage chronic spinal pain, offering longer-lasting relief than glucocorticoids (Becker et al., 2007; Buchheit et al., 2023; Godek et al., 2023; Goni et al., 2015).

Objective monitoring of such interventions remains challenging because treatment response in CES is traditionally judged by subjective clinical assessment and owner questionnaires. Wearable inertial measurement unit (IMU) sensors detect changes in gait parameters, such as swing and stance phases or joint extension and flexion, in dogs with hip dysplasia (Blättler et al., 2024). These systems enable objective gait analysis outside specialized motion laboratories, with limb-mounted IMUs identifying temporal gait events in strong agreement with pressure-sensor walkways (Zhang et al., 2022). Field accelerometry has been similarly used to characterize canine gait patterns, including stride frequency and acceleration profiles (Hayati et al., 2019). IMUs quantify kinematic and temporal gait characteristics, rather than replace kinetic force-plate measurements or validated markers of pain relief (Clark et al., 2014; Hayati et al., 2019; Zhang et al., 2022).

This retrospective study had two objectives: to determine whether IMU-based analysis is feasible for objective, longitudinal monitoring of limb function in dogs with CES under routine clinical conditions, and to describe exploratory changes in gait parameters following a single combined epidural injection of ACS and triamcinolone acetonide. We hypothesized that this combined intervention would be associated with measurable, directional changes in limb gait parameters over time.

## 2 Materials and methods

### 2.1 Patient selection and eligibility criteria

This retrospective study evaluated medical records and clinical examinations of dogs presented to a single veterinary practice (orthoVET, Frenkendorf, Switzerland) for orthopedic assessment between 2019 and 2024. Included records comprised patients with a pain-dominant phenotype of CES secondary to suspected DLSS or neuroforaminal stenosis and had previously received a concurrent epidural injection of ACS and GC. Dogs were referred for clinical signs originating from the pelvic limbs or lumbosacral spine, including lumbar pain, difficulty rising, reduced physical performance, hindlimb weakness, kyphotic posture, pelvic asymmetry, abnormal gait patterns, reluctance to climb stairs, or difficulty jumping into vehicles.

All dogs underwent standardized visual gait assessment (recorded on video), a thorough orthopedic examination (focusing on the shoulder girdle, pelvic girdle, and spine), and a full neurological examination (including proprioception and flexor reflexes). To isolate a pain-dominant CES cohort resulting from DLSS or neuroforaminal stenosis, records were excluded if dogs exhibited concurrent orthopedic disorders (e.g., cranial cruciate ligament rupture, stifle pathology, hip dysplasia, elbow dysplasia, or distal limb abnormalities) or overt neurological deficits (e.g., ataxia, paresis, or absent reflexes). Mild or inconsistent reflex changes were not grounds for exclusion. Dogs with previous lumbosacral surgery were eligible if presenting with clinical pain relapse. Clinical, orthopedic, and neurological evaluations were performed by a single experienced clinician.

### 2.2 Diagnostic imaging of the lumbosacral region

Spinal radiographs were obtained in all included cases in a standardized standing position (Shimadzu MobileDaRt Evolution MX8, Canon CXDI-710C Wireless) to document degenerative lumbosacral changes under physiological, load-bearing conditions. A subset of study population (50%) also underwent advanced diagnostic imaging, including computed tomography (CT), and/or magnetic resonance imaging (MRI), when clinically indicated.

### 2.3 Processing of Orthogen-ACS

Orthogen-ACS was prepared using a commercially available Orthogen Device vet (Orthogen Veterinary GmbH, Düsseldorf, Germany) according to the manufacturer’s instructions. Fasted dogs underwent jugular venipuncture (21G butterfly cannula) under aseptic conditions without sedation, yielding 15 mL of whole blood directly into a sterile Orthogen Device vet (Fig. 1A). The device was immediately incubated upright at 37°C for 3–6 h (MF-6W, HCP-Technology, Nortrup, Germany) to induce extended coagulation (Fig. 1B). Following incubation, the device was centrifuged in a swing-out rotor (Z306, Hermle, Universal Centrifuge, Wehingen, Germany) at 1500 *× g* for 3 minutes (Fig. 1C), yielding approximately 5 mL of Orthogen-ACS, automatically filtered into a sterile glass vial (Fig. 1D–F). The serum was administered immediately or frozen at −18°C in a sealed vial (Steri-Tamp, Allied Pharmacy Products, Delray Beach, United States). Frozen vials were thawed overnight at 5°C before use.

**Fig. 1:**
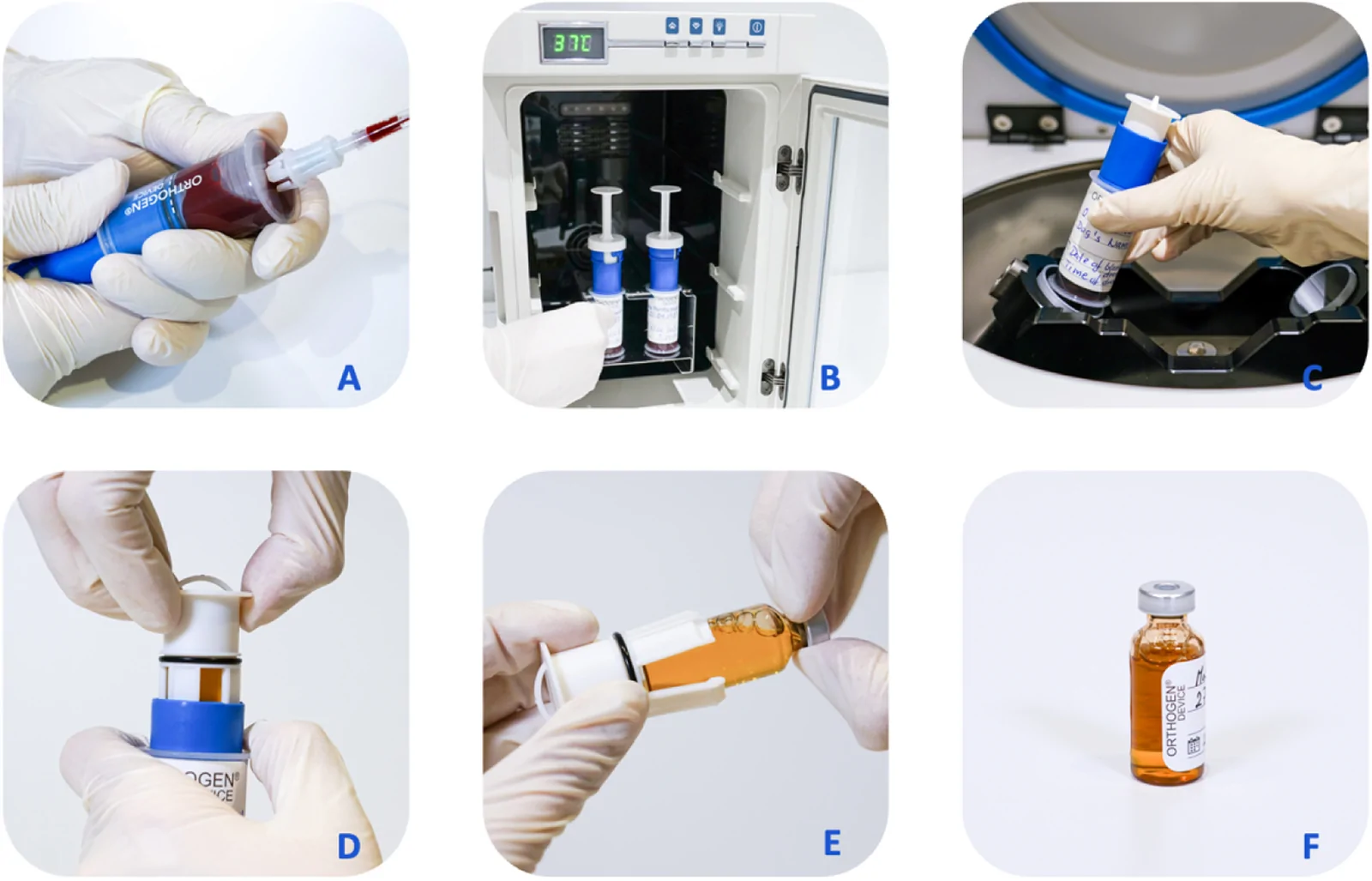
Orthogen-ACS processing steps: (**A)** Blood collection using the Orthogen Device vet; (**B)** Incubation at 37°C; (**C)** Centrifugation; (**D–F)** Retrieval of processed Orthogen-ACS in the sterile vial.

### 2.4 Epidural infiltration protocol

Epidural injections were performed in all patients using a standardized aseptic technique. Prior to sedation, a peripheral intravenous catheter was placed. To ensure muscle relaxation and mobility, dogs received intravenous sedation with medetomidine hydrochloride (2 µg/kg; Dorbene vet, 1 mg/mL, Dr. E. Graeub AG, Switzerland) and butorphanol tartrate (0.3 mg/kg; Morphasol, 10 mg/mL, Dr. E. Graeub AG, Switzerland), followed by propofol (2 mg/kg; Propovet, Zoetis Schweiz GmbH, Switzerland). Endotracheal intubation and isoflurane maintenance were used when clinically indicated based on patient age or physical status.

Patients were positioned in lateral or sternal recumbency with hindlimbs flexed cranially. The lumbosacral region (L7–S1) was clipped, aseptically prepared, and covered with an antimicrobial incise drape (3M™ Ioban™ 2, 3M Deutschland GmbH, Germany) and fenestrated surgical drape. Anatomical landmarks (iliac crest, L7 dorsal spinous process, and L7–S1 intervertebral space) were identified by palpation. A spinal needle (20–21G × 2” for small dogs; 21–22G × 3” for medium/large dogs) was advanced along the midline perpendicular to the dorsal plane through the supraspinous ligament and ligamentum flavum until entering the epidural space. Correct needle placement was confirmed radiographically in all cases (Fig. 2).

**Fig. 2:**
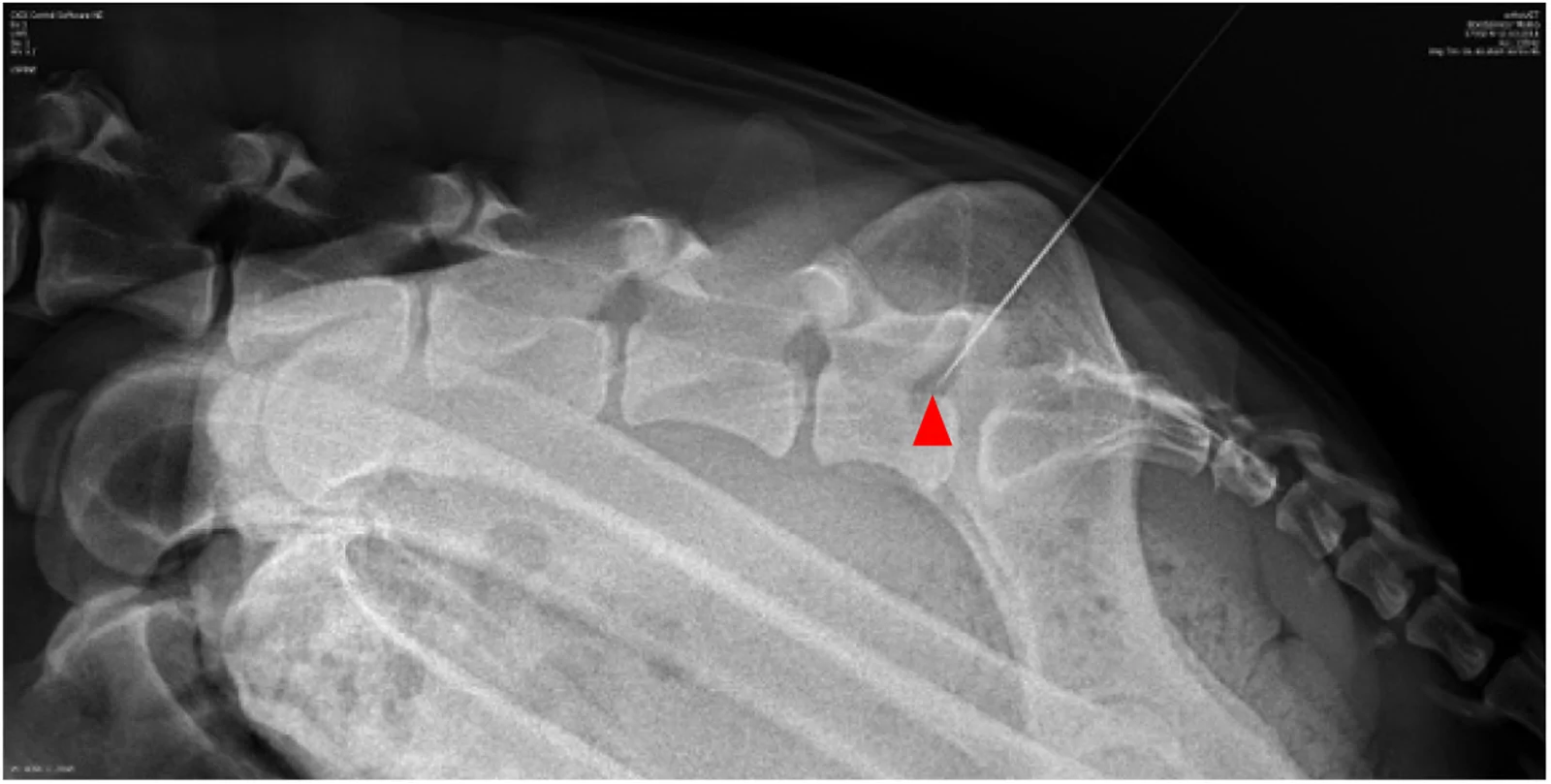
Radiographic confirmation of needle placement in the lumbosacral epidural space (lateral view; arrow indicates needle tip).

Orthogen-ACS (3–4 mL) was injected slowly into the epidural space, immediately followed by triamcinolone acetonide (0.5 mg/kg; Kenacort-A 10, 10 mg/mL, Dermapharm AG, Switzerland). Patients were monitored under supervision until full recovery.

### 2.5 Objective gait analysis using the LupoGait system

Gait was assessed using the LupoGait, a wearable, inertial measurement unit (IMU)-based motion analysis system (4Dvets AG, Switzerland), designed for field evaluation of quantitative canine kinematics. All assessments were operated by the same trained veterinary assistant in the presence of the owner.

#### 2.5.1 Sensor configuration

The LupoGait system comprises six wireless IMU sensors equipped with tri-axial accelerometers and gyroscopes to record linear acceleration and angular velocity during walking. These data enable quantitative extraction of spatiotemporal and symmetry-related gait parameters using validated proprietary algorithms (Altermatt et al., 2023). Sensors were secured using Velcro straps within a fitted jacket and positioned bilaterally over the proximal forelimbs (elbow region), hindlimbs (stifle region), sacrum (laterally), and scapulae (dorsally along the midline). This configuration recorded movement patterns and motion of limbs and trunk during locomotion (Fig. 3).

**Fig. 3:**
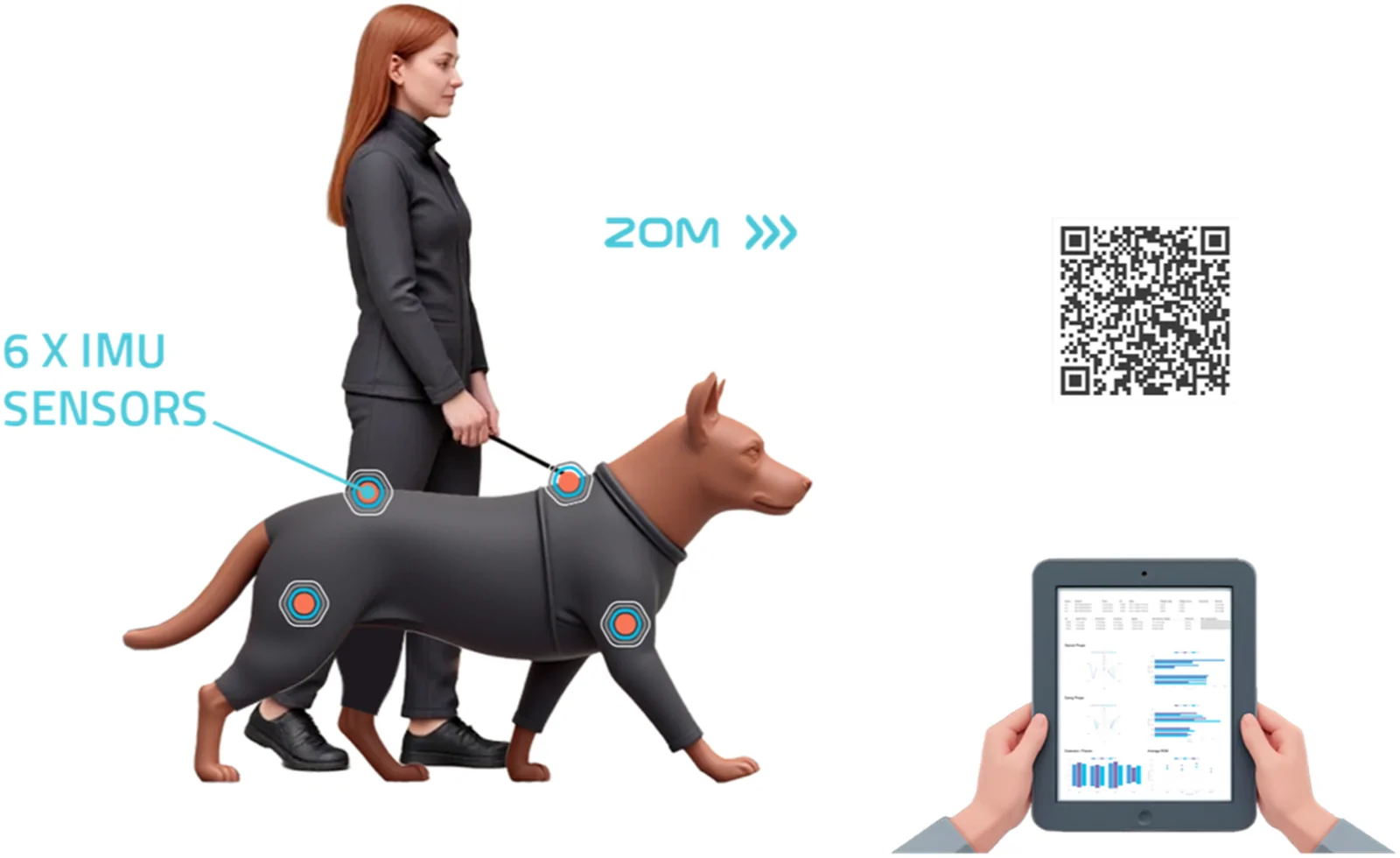
Sensor configuration of the six-sensor inertial measurement unit LupoGait system. For a complete demonstration, scan the QR code.

#### 2.5.2 Measurement protocol

Gait assessments were conducted outdoors on a flat, 20 m asphalt track. Dogs walked at a steady pace without stopping or pulling. Measurements were performed prior to sedation (baseline) and repeated at the follow-up under an identical protocol.

Body weight and withers height were recorded before each walking sequence to normalize gait parameters and to classify dogs into subgroups based on body size. Sensor calibration was performed during two 5 s static intervals immediately before and after each walking trial. Sensor data were transmitted to the LupoGait analysis platform for data processing (Altermatt et al., 2023).

#### 2.5.3 Evaluated gait parameters

Accelerometer and gyroscope signals provided the basis for gait analysis. From these data, a predefined set of clinically relevant gait parameters was computed, serving as IMU-derived kinematic and spatiotemporal descriptors for an individual evaluation over time.

Six parameter categories were evaluated:

• Step and stride frequency
• Stance and swing phase ratio
• Forelimb and hindlimb range of motion (ROM)
• Left-right limb symmetry indices
• Flexion and extension movement profiles
• Vertical dynamic proxies (IMU-derived load parameters)

Parameters were compared between baseline and follow-up measurements to evaluate longitudinal gait changes after treatment. Although limb-mounted IMUs can accurately identify canine swing and stance timing relative to pressure-walkways, LupoGait outputs were not validated against a pressure walkway, force plate, or optoelectronic motion-capture system in this study (Zhang et al., 2022). Consequently, all derived variables were interpreted strictly as IMU-based kinematic descriptors rather than direct kinetic force measurements or validated markers in pain relief.

#### 2.5.4 Clinical follow-up

Follow-up gait measurements were performed 3–4 weeks post-injection, based on owner availability. Evaluation included repeat visual gait analysis, orthopedic examination, neurological assessment, and owner questionnaire adapted from the Canine Brief Pain Inventory (CBPI; Brown et al., 2007). Owner questionnaire responses were reviewed qualitatively to provide clinical information but were not converted to validated severity scores or analyzed statistically as direct evidence of efficacy.

### 2.6 Statistical analysis

Gait metrics were averaged across forelimb and hindlimb pairs to generate single pre- and post-treatment values per dog. Continuous variables are presented as median and interquartile range (IQR, 25th–75th percentile). Normality of paired differences was evaluated using the Shapiro–Wilk test. Because paired differences violated normality, non-parametric Wilcoxon signed-rank test was used for paired comparisons. The magnitude and precision of changes were expressed as Hodges–Lehmann (HL) median paired differences with 95% confidence intervals (CIs). Effect sizes were calculated using the matched-pair rank-biserial correlation (*r*). Statistical significance was defined as a two-sided *p* < 0.05. To control type I error across multiple secondary gait parameters, Benjamini–Hochberg false-discovery-rate (FDR) corrections were applied to whole-group comparisons.

The hindlimb stance phase ratio was designated as the primary outcome measure, as it represents a clinically established kinematic index of weight-bearing relevant to lumbosacral pathology. All other parameters were evaluated as secondary or exploratory. Because this was a retrospective analysis, the designation was made for the present study. Exploratory subgroup analyses (age, body weight, sex, and neuter status) were performed for hypothesis generation. Given small subgroup sizes, *p*-values were unadjusted for multiple testing. As a retrospective study, no *a priori* power calculation was performed. A post-hoc sensitivity analysis was conducted to establish the minimal detectable effect size given the sample size. Statistical analyses and visualization were performed using Microsoft Excel, jamovi (v2.7, on R v4.5), Python 3.12, and Matplotlib.

## 3 Results

A total of 44 dogs were included in the retrospective analysis. Six dogs (13.6%) had undergone previous lumbosacral surgery and were included following clinical pain relapse. None of the dogs showed marked neurological deficits, and abnormalities were limited to mild, inconsistent reflex changes. Follow-up clinical evaluations and unvalidated owner-reported questionnaires provided qualitative context for kinematic findings but were excluded from statistical analysis and do not represent evidence of treatment efficacy.

Radiological evaluation confirmed degenerative spinal alterations in all 44 dogs. Advanced cross-sectional imaging (CT and/or MRI) was available for a subset of 22 dogs (50%) with imaging findings consistently matching the anatomical location of clinical pain. Lesions were monosegmental or multisegmental, characterized predominantly by spondyloarthropathy deformans with or without evidence of central disc protrusion, alongside concomitant facet joint degeneration. Load-bearing standing radiographs demonstrated clear degenerative alterations in the lumbosacral region (Fig. 4A–C), specifically highlighting the L7–S1 neural foramen, transitional anatomy, and sacroiliac joints.

**Fig. 4:**
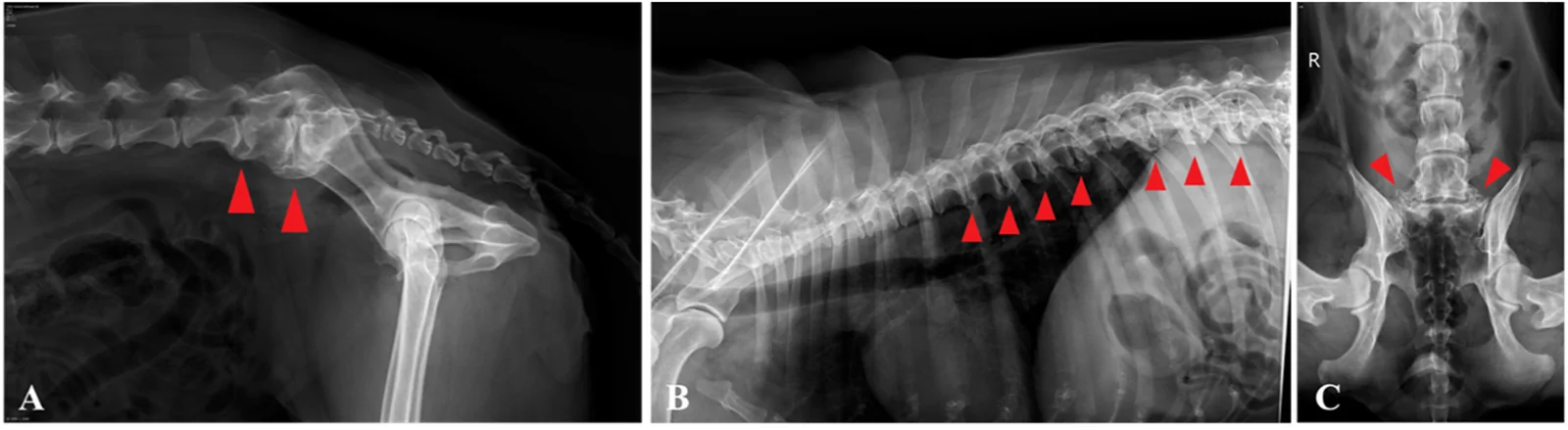
Standing radiographic images showing initial degenerative alterations in the cauda equina region (indicated by arrows). (**A)** Lumbosacral cauda equina region in standing position. (**B)** Thoracic spine showing degenerative spondyloarthropathy deformans. (**C)** Pelvis demonstrating bilateral neural foraminal osseous changes.

In unadjusted comparisons, several hindlimb kinematic metrics showed nominal changes post-treatment (Fig. 5A–D). However, none of these changes retained statistical significance after Benjamini-Hochberg FDR correction across cohort. For the primary outcome measure, the median hindlimb stance phase increased from 0.21 (IQR 0.15–0.26) to 0.22 (IQR 0.18–0.28, HL +0.03, 95% CI 0.005 to 0.05, *p* = 0.018) (Fig. 5A).

**Fig. 5:**
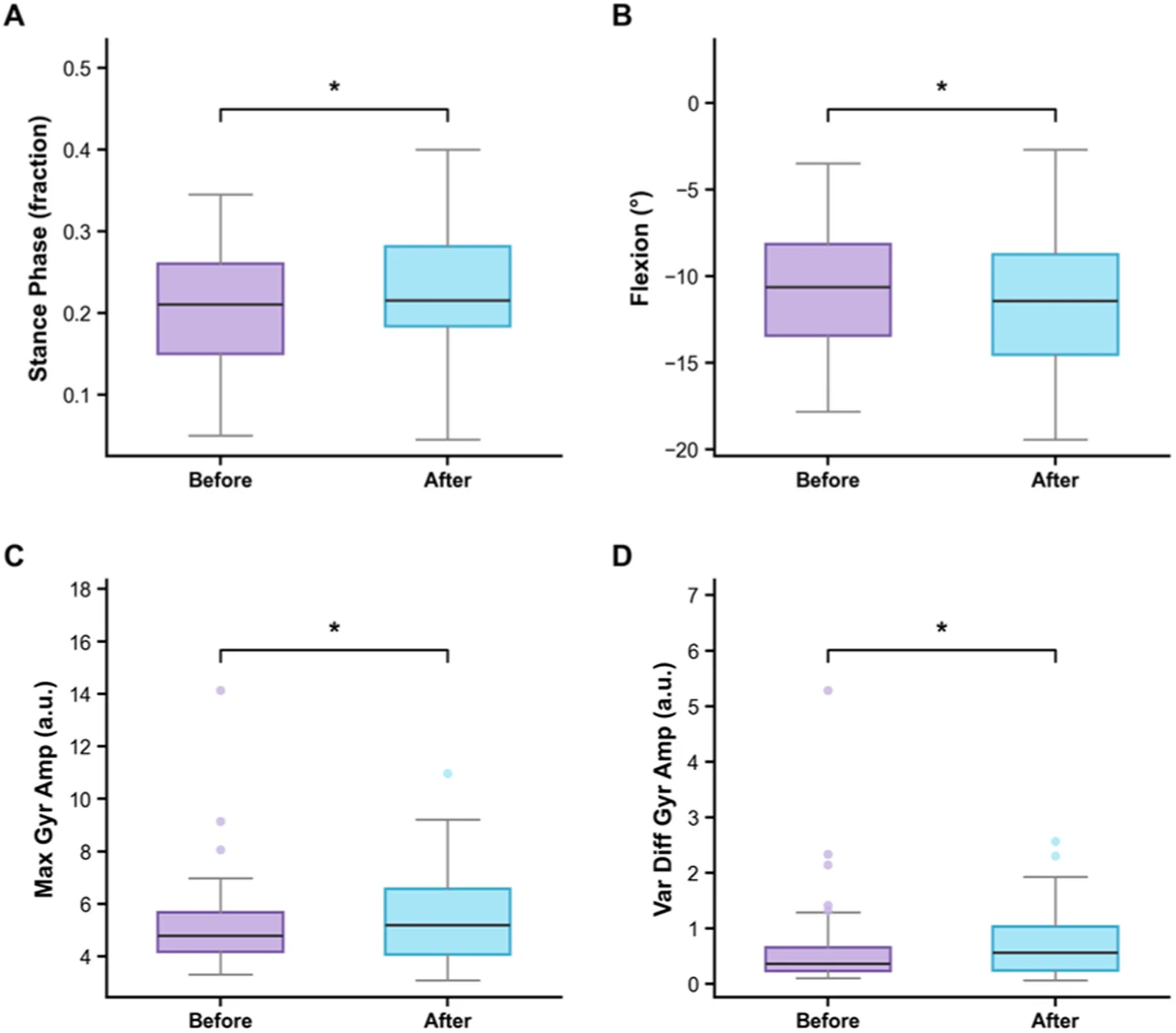
Hindlimb kinematic parameters before (purple) and after (blue) epidural autologous conditioned serum and glucocorticoid infiltration in dogs with cauda equina syndrome (*n* = 44). **(A)** Stance phase ratio (primary outcome, fraction of the stride). **(B)** Flexion angle (°). **(C)** Maximum gyroscope amplitude (arbitrary units, a.u.). **(D)** Variance of differentiated gyroscope signal (a.u.). Asterisks (*) denote nominal pre- to post-treatment unadjusted differences (Wilcoxon signed-rank test *p* < 0.05; false-discovery-rate *p* ≥ 0.05).

Among secondary hindlimb parameters, nominal unadjusted increases were observed in maximum gyroscope amplitude (from 4.77 [IQR 4.16–5.67] to 5.18 [IQR 4.07–6.57, HL +0.32, 95% CI 0.02 to 0.69, *p* = 0.032]) and flexion angle magnitude (shifting to more negative value from −10.63 [IQR −13.45 to −8.17] to −11.45 [IQR −14.54 to −8.75, HL −0.64, 95% CI −1.33 to −0.07, *p* = 0.029]) (Fig. 5B). Signal variance also showed an unadjusted increases for the differentiated gyroscope signal (from 0.357 [IQR 0.228–0.656] to 0.554 [IQR 0.238–1.03, HL +0.10, 95% CI 0.01 to 0.23, *p* = 0.036]) and angular velocity perpendicular to the joint axis (from 1.50 [IQR 0.95–2.08] to 1.85 [IQR 1.12–3.00, HL +0.30, 95% CI 0.02 to 0.68, *p* = 0.037]).

Other secondary hindlimb parameters showed no unadjusted differences pre- to post- treatment. Hindlimb ROM increased from a median of 27.30 (IQR 20.93–32.26) to 28.87 (IQR 21.34–33.13; HL +1.01, 95% CI −0.06 to 2.20, *p* = 0.064). Mean gyroscope amplitude (HL +0.11, 95% CI −0.02 to 0.23, *p* = 0.126), mean differentiated gyroscope amplitude (HL −0.007, 95% CI −0.017 to 0.002, *p* = 0.144), and median extension remained unchanged (12.24 to 12.79; HL +0.40, 95% CI −0.30 to 1.04, *p* = 0.258). All kinematic metrics, CI, and effect sizes are summarized in Table 1.

**Table 1:** Hindlimb kinematic parameters before and after combined autologous conditioned serum and glucocorticoid epidural injection.

Parameter	Before, median (IQR)	After, median (IQR)	HL estimate (95% CI)	Wilcoxon *p*	Effect size *r*
Stance phase (fraction of the stride)	0.21 (0.15 to 0.26)	0.22 (0.18 to 0.28)	+0.03 (0.005 to 0.05)	**0.018**	0.36
Flexion (°)	−10.63 (−13.45 to −8.17)	−11.45 (−14.54 to −8.75)	−0.64 (−1.33 to −0.07)	**0.029**	−0.33
Maximum gyroscope amplitude (a.u.)	4.77 (4.16 to 5.67)	5.18 (4.07 to 6.57)	+0.32 (0.02 to 0.69)	**0.032**	0.32
Variance of differentiated gyroscope signal (a.u.)	0.357 (0.228 to 0.656)	0.554 (0.238 to 1.03)	+0.10 (0.01 to 0.23)	**0.036**	0.31
Variance of angular velocity perpendicular to the joint axis (a.u.)	1.50 (0.95 to 2.08)	1.85 (1.12 to 3.00)	+0.30 (0.02 to 0.68)	**0.037**	0.31
Average ROM (°)	27.30 (20.93 to 32.26)	28.87 (21.34 to 33.13)	+1.01 (−0.06 to 2.20)	0.064	0.28
Mean gyroscope amplitude (a.u.)	2.53 (2.25 to 2.94)	2.72 (2.14 to 3.23)	+0.11 (−0.02 to 0.23)	0.126	0.23
Mean differentiated gyroscope amplitude (a.u.)	−0.079 (−0.093 to −0.061)	−0.090 (−0.126 to −0.066)	−0.007 (−0.017 to 0.002)	0.144	−0.22
Extension (°)	12.24 (8.50 to 14.08)	12.79 (9.11 to 14.50)	+0.40 (−0.30 to 1.04)	0.258	0.17

Note: a.u. – arbitrary units; ROM – range of motion; IQR – interquartile range; CI – confidence interval.

Forelimb kinematic parameters were largely unaffected. Gyroscope correlation fell from median of 0.003 (IQR −0.027 to 0.055) to −0.008 (IQR −0.059 to 0.048, HL −0.022, 95% CI −0.046 to −0.0002, *p* = 0.049). Forelimb maximum gyroscope amplitude remained unchanged (HL +0.10, 95% CI −0.14 to 0.35, *p* = 0.42).

Hindlimb ROM correlated positively with extension before and after treatment (*r* = 0.93 and *r* = 0.89, both *p* < 0.001) and negatively with flexion (*r* = −0.92 at both timepoints, *p* < 0.001) (Fig. 6A–D). Extension and flexion were inversely correlated (*r* = −0.81 before, *r* = −0.75 after, both *p* < 0.001). Before-after self-correlations were highest for ROM (*r* = 0.81), flexion (*r* = 0.78), extension (*r* = 0.67), and stance phase ratio (*r* = 0.48, all *p* < 0.001).

**Fig. 6:**
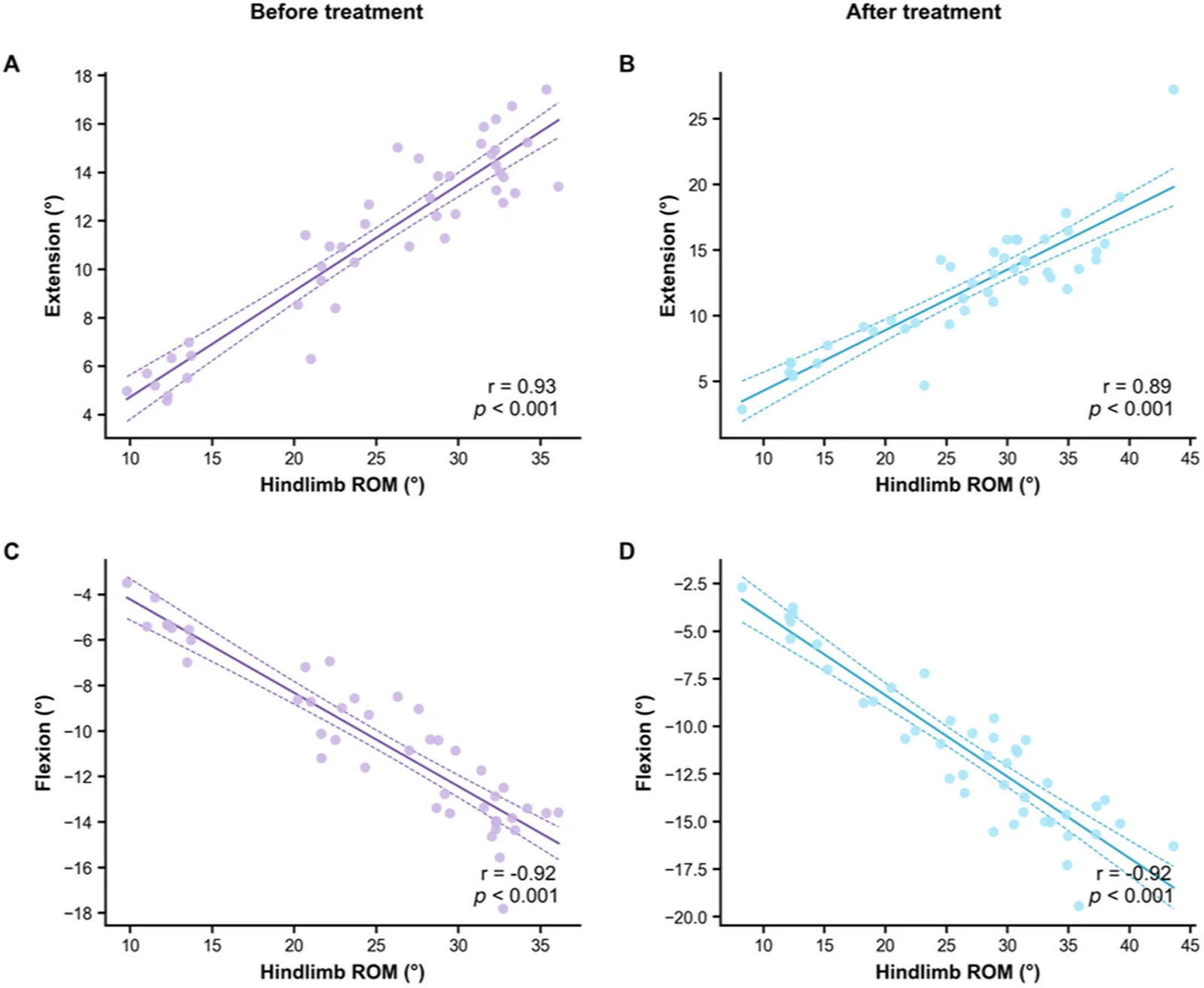
Individual dog correlations between hindlimb range of motion (ROM) and joint-angle parameters before (A, C) and after (B, D) combined epidural autologous conditioned serum and glucocorticoid infiltration (*n* = 44). **(A)** Baseline correlation between ROM and extension. **(B)** Post-treatment correlation between ROM and extension. **(C)** Baseline correlation between ROM and flexion. **(D)** Post-treatment correlation between ROM and flexion.

### 3.1 Exploratory subgroup analyses

Exploratory subgroup analyses across sex, neutering status, age, and body weight categories (Table 2) revealed nominal parameter shifts. However, no subgroup comparison retained statistical significance. Because subgroups were small (5–28 dogs), these analyses were unadjusted and remained underpowered and hypothesis-generating. A sensitivity analysis indicated that, while the whole cohort (*n* = 44) achieved 80% power to detect a moderate standardized effect size (0.44), individual subgroups lacked statistical power to detect minor kinematic changes.

**Table 2:** Characteristics and subgroups of dogs.

Variables	Dogs (*n* = 44)
Weight, kg	
10–24, *n* (%)	11 (25)
25–39, *n* (%)	28 (63.6)
≥40, *n* (%)	5 (11.4)
Age, y/o	
Young 0–5, *n* (%)	6 (13.6)
Adult 6–10, *n* (%)	27 (61.4)
Senior ≥11, *n* (%)	11 (25)
Female (%)	21 (47.7)
Male (%)	23 (52.3)
Neutered (%)	25 (56.8)
Intact (%)	19 (43.2)

Where nominal, unadjusted differences were observed primarily in hindlimb parameters among large dogs (*n* = 28). Directional patterns differed by sex and neuter status: spayed females showed unadjusted increase in hindlimb gyroscope amplitude and forelimb symmetry, whereas castrated males showed unadjusted decrease in symmetry and forelimb energy metrics. Senior and giant breed subgroups showed no nominal changes. Full kinematic metric changes, CI, and significance levels are detailed in Supplementary Table S1.

## 4 Discussion

### 4.1 Principal findings

This retrospective study evaluated whether a wearable IMU-based motion analysis system could detect kinematic changes in dogs with CES following epidural administration of ACS and triamcinolone acetonide. The principal finding was a modest unadjusted increase in the hindlimb stance phase ratio. Spatiotemporal metrics, particularly stance phase ratio, are established indicators for characterizing hindlimb lameness in dogs (Park et al., 2024). Although an increased stance phase ratio is associated with altered weight-bearing, none of the evaluated metrics remained statistically significant following FDR correction across the cohort (Aristizabal Escobar et al., 2017; Sutton et al., 2016).

Secondary hindlimb parameters showed a nominal unadjusted increase in flexion excursion, aligning with sagittal-plane joint mobility changes (Headrick et al., 2014), alongside increases in gyroscope-derived measures of angular velocity and variance. Although these parameter shifts suggest localized changes in pelvic limb rotational dynamics, IMU-derived gyroscope metrics have not been validated as direct clinical biomarkers of pain relief or neurological recovery in canine CES. In contrast, forelimb parameters were mostly unchanged, limited to a minor unadjusted reduction in gyroscope correlation without changes in amplitude. While this isolated finding requires cautious interpretation, given the loss of significance after FDR correction, wearable IMU systems are recognized for their sensitivity in capturing subtle gait variations (Blättler et al., 2024).

The predominant pattern of kinematic variation within the pelvic limbs aligns biologically with the lumbosacral localization of CES (Meij & Bergknut, 2010). Epidural infiltration of anti-inflammatory agents into the lumbosacral space aims to modulate local inflammatory cascades, which theoretically facilitates altered pelvic limb motion (De Risio et al., 2001; Suwankong et al., 2008). However, because of the uncontrolled retrospective design and loss of statistical significance after FDR adjustment, these findings cannot confirm treatment efficacy or isolate the specific contribution of ACS. While these exploratory findings cannot establish therapeutic benefit, the nominal kinematic patterns provide helpful context for concurrent ACS and GC administration in dogs with CES. Furthermore, wearable IMU motion analysis detected subtle gait shifts (Sutton et al., 2016), establishing an objective baseline to power future prospected, placebo-controlled trials.

### 4.2 Preliminary insights

Several IMU-derived parameters require cautious clinical interpretation. While gyroscope-derived measures of angular velocity and variance provide objective, quantitative descriptors of limb motion, their diagnostic validity of direct pain markers in dogs with CES remains unestablished (Engelsman et al., 2022; Jenkins et al., 2018; Ladha et al., 2017). The strong correlation between ROM and sagittal joint angles (extension and flexion) is mathematically expected, as ROM is derived from the extension-flexion excursion (Pinna et al., 2021). Combined with strong baseline-to-follow-up self-correlations, these relationships serve as internal consistency checks across sensor channels, demonstrating IMU-based motion system’s signal stability.

Exploratory comparison across sex, neuter status, age, and body weight yielded no statistically significant changes. Minor unadjusted hindlimb variations in larger dogs reflect higher statistical power within this cohort. Unadjusted variations across neuter status align with morphometric and gonadal influences on canine locomotion rather than injection-based differences (Della Valle et al., 2022). However, they require confirmation in larger, prospectively powered studies.

### 4.3 Combined ACS and glucocorticoid therapy

Epidural infiltration is an established conservative treatment option for degenerative lumbosacral stenosis (DLSS) with corticosteroid infiltration alone reported to achieve clinical improvement in up to 79% of dogs with Hansen type II lumbosacral protrusion (Janssens et al., 2009). The growing interest in autologous orthobiologics reflects a broader shift toward disease-modifying, inflammation-resolving strategies to complement traditional anti-inflammatory therapy.

In veterinary orthopedics, autologous blood products, such as leukocyte-poor platelet rich plasma (PRP) have demonstrated gait improvements by concentrating platelets and growth factors while reducing pro-inflammatory leukocyte content (Hernández-Guerra et al., 2021). ACS advances this concept by providing completely cell-free secretome generated through controlled extracorporeal blood incubation. This process stimulates the release of key bioactive mediators, including IL-1Ra, IL-10, TGF-β1, and VEGF alongside mitochondrial-derived factors, extracellular vesicles, proteins, lipids, and mitochondria (Hang et al., 2024; Sawyere et al., 2016; Soontararak et al., 2022). These secretome components collectively modulate IL-1β-driven catabolism, reduce NF-κB activation, and support extracellular matrix synthesis (Baltzer et al., 2009; Frizziero et al., 2013; Wehling et al., 2007). From a practical and safety perspective, removal of cellular components and coagulation factors (fibrinogen and prothrombin) via centrifugation and sterile filtration (0.22 *µm*) minimizes local inflammatory reactions and infection risks associated with non-filterable cellular injectates (Alves et al., 2022a; Arita & Tobita, 2024). Automated processing and cryopreservation overcome the technician-dependent variability and shelf-life constraints typical of PRP systems, positioning ACS as a highly standardized, practical orthobiologic for epidural administration (Carvalho et al., 2024; Roth et al., 2025; Zhou et al., 2025).

The biological rationale for combining ACS with long-acting glucocorticoid lies in potential multimodal synergy: corticosteroids provide rapid, short-term anti-inflammatory relief, while ACS supports sustained, long-term inflammation resolution and tissue homeostasis (Alves et al., 2022b; Evans et al., 2016; Godek et al., 2023; Wehling et al., 2007). In the retrospective medical records evaluated here, owner reports and re-examiner frequently noted increased patient activity and functional improvements. However, because these subjective assessments relied on unvalidated, unblinded, and underscored adaptation of CBPI, observations must be interpreted strictly as informal qualitative impression rather than standardized evidence of analgesia.

### 4.4 IMU-based kinematic evaluation

While force-plate and instrumented treadmill systems represent the reference standard for kinetic gait analysis in dogs, their technical, logistical, and infrastructural constraints severely restrict their feasibility in routine clinical practices and field-based studies (Amimoto et al., 2020; Ballagas et al., 2004; Manupibul et al., 2023; Söhnel et al., 2022). Wearable IMU-based systems, such as LupoGait, overcome these practical limitations by enabling objective motion tracking under standard clinical conditions without the time-consuming calibration procedures required by conventional optical kinematics (Altermatt et al., 2023; Sandberg et al., 2020; Stenman, 2019; Ribera d'Alcalà et al., 2023).

In this study, LupoGait-derived variables were used as within-subject proxies for movement and compared longitudinally across follow-up intervals. These metrics were not cross-validated against a force plate or kinetic reference standard within this canine cohort. Although accelerometer-derived signals in human biomechanics can estimate peak vertical ground forces (PVF) with narrow error margins, veterinary applications require greater caution (Bach et al., 2022; Pimentel et al., 2024; Wang et al., 2024; Zhu et al., 2026). Trunk-mounted accelerometer studies in dogs demonstrate only moderate agreement with force-platform analysis for PVF, showing weak reliability for individual hindlimb measurements when sensors are positioned over the lumbar spine (Clark et al., 2014).

Accordingly, vertical dynamic outputs from the LupoGait were interpreted as indirect movement descriptors rather than direct force-plate-equivalent PVF measurements. By avoiding kinetic overreach, this methodology highlights the primary strengths of wearable IMU technology in canine CES: providing standardized, repeatable, observer-independent motion profiling suitable for longitudinal clinical monitoring under real-world conditions.

### 4.5 Future directions in gait analysis

Expanding beyond six-sensor configuration used in this study is a logical next step for canine gait analysis. A twelve-sensors setup would capture detailed, segment-specific motion data across key lumbosacral regions: L5, sacrococcygeal junction, and major limb joints (elbow, shoulder, hip, and stifle). Because the affected lumbosacral nerve roots (L5–S3) control pelvic limb motor function, precise tracking would allow clinicians to better monitor therapeutic interventions, including surgery, epidural infiltrations, pharmacological treatments, and physiotherapy (American Association of Neurological Surgeons, 2024).

Additionally, a twelve-sensor system would facilitate evidence-based assessment of hindlimb lameness resulting from neuroforaminal degeneration. By capturing complex functional tasks, such as rising from a resting position, developing propulsive force, and ascending stairs, quantifying these dynamic movements would deepen our biomechanical understanding of lumbosacral pathophysiology and support objective evaluation of therapeutic outcomes.

### 4.6 Limitations and conclusions

Several limitations warrant cautious interpretation of these findings. First, this was a short, retrospective, uncontrolled before-after study with a small sample size, making it susceptible to regression to the mean and natural disease fluctuations. Second, because ACS was administered with GC, treatment-specific effects of ACS cannot be isolated. Third, evaluator blinding was absent, introducing potential assessment bias and advanced diagnostic imaging (CT and/or MRI) was performed in only 50% of the study population. Fourth, the cohort primarily represented a pain-dominant DLSS and neuroforaminal stenosis phenotype rather than dogs with substantial neurological deficits. Finally, the study was underpowered to detect small effect sizes, and no evaluated parameters remained statistically significant after correction for multiple comparisons, making all observed changes preliminary and exploratory.

In conclusion, although the lack of post-correction statistical significance precludes definitive causal conclusions regarding treatment efficacy, these findings reveal directionally coherent pelvic-limb kinematic trends following combined ACS and GC epidural injection. However, prospective, randomized, and placebo-controlled trials are required to isolate and confirm treatment-specific benefits across diverse patient characteristics. Nevertheless, this study offers preliminary support for IMU-based gait analysis as a practical, objective tool for longitudinal kinematic monitoring under routine clinical conditions.

## Ethics declaration

This study was conducted in accordance with the ARRIVE (Animal Research: Reporting of In Vivo Experiments) guidelines. This study was approved by the Amt für Lebensmittelsicherheit und Veterinärwesen - Department für Kleintiere. (Approval No. 33110 - BL/515/2020).

### Ethical statement

This manuscript matches the principles of the Committee on Publication Ethics. Furthermore, the involved use of animal subjects is according with the NC3Rs ARRIVE Gudelines.

## CRediT authorship contribution statement

**Patrick Blaettler Monnier:** Writing – original draft, Validation, Resources, Methodology, Investigation, Formal analysis, Data curation, Conceptualization. **Indre Matsuzaki:** Writing – original draft, Visualization, Validation, Methodology, Formal analysis. **Nico Gruetter:** Project administration, Formal analysis. **Julien Troillet:** Writing – review & editing, Supervision, Project administration, Methodology, Conceptualization.

## Declaration of competing interest

One author is respectively co-founder and shareholder of 4DVets AG, the manufacturer of the discussed system. All authors worked together on a research project financed by Orthovet, Fasanenstrasse 13, CH-4402 Frenkendorf.
