# Supplementary material for: Kinematic changes following concurrent epidural injection with autologous conditioned serum (ACS) and glucocorticoids in dogs with cauda equina syndrome
Source: Vet Anim Sci. 2026 Oct 20;34:100911. doi: 10.1016/j.vas.2026.100911 (PMC13735292; doi:10.1016/j.vas.2026.100911)
Supplement: Supplementary file 1 [file mmc1.docx]

**Supplementary Material**

**Supplementary Table S1**

*Subgroup analyses summary significant results (p < 0.05)*

| **Subgroup** | **Limb** | **Parameter** | **HL (95% CI)** | **Wilcoxon *p*** | **Effect *r*** |
| --- | --- | --- | --- | --- | --- |
| **Female** | Hind | Flexion | −0.975 (−2.1 to −0.19) | 0.0195 | −0.5 |
|  | Hind | Maximum gyroscope amplitude | +0.429 (+0.0089 to +0.97) | 0.046 | +0.44 |
| **Male** | Hind | Stance Phase | +0.0437 (+0.017 to +0.065) | 0.0062 | +0.57 |
|  | Hind | Energy of acceleration | +6.06 (+0.96 to +12) | 0.0254 | +0.46 |
|  | Hind | Swing Phase | −0.04 (−0.072 to −0.005) | 0.0296 | −0.47 |
|  | Front | Vertical force proxy, IMU-derived | −190 (−3.8e+02 to −9.7) | 0.0354 | −0.44 |
|  | Hind | Normalized step length | +0.067 (+0.0021 to +0.31) | 0.0449 | +0.42 |
|  | Hind | Step Length | +0.0398 (+0.00025 to +0.14) | 0.0484 | +0.41 |
| **Neutered** | Hind | Symmetry PR | −0.827 (−1.7 to −0.21) | 0.0115 | −0.5 |
|  | Hind | Maximum gyroscope amplitude | +0.479 (+0.093 to +0.89) | 0.0125 | +0.49 |
|  | Hind | Variance ang. vel. joint axis | +0.407 (+0.064 to +0.83) | 0.0187 | +0.47 |
|  | Front | Normalized energy of acceleration | −0.155 (−0.31 to −0.04) | 0.0203 | −0.46 |
|  | Hind | Flexion | −1.12 (−2.2 to −0.15) | 0.0219 | −0.45 |
|  | Hind | Mean gyroscope amplitude | +0.173 (+0.019 to +0.36) | 0.0296 | +0.43 |
|  | Hind | Median gyroscope amplitude | +0.161 (+0.015 to +0.32) | 0.0342 | +0.42 |
| **Spayed female** | Front | Symmetry AR | +1.17 (+0.11 to +2.1) | 0.0215 | +0.59 |
|  | Hind | Mean gyroscope amplitude | +0.235 (+0.00095 to +0.47) | 0.0479 | +0.51 |
|  | Hind | Median gyroscope amplitude | +0.213 (+0.01 to +0.39) | 0.0479 | +0.51 |
|  | Front | Symmetry BF | +1.49 (+0.08 to +3) | 0.0479 | +0.51 |
| **Castrated male** | Hind | Symmetry PL | −1.47 (−2.3 to −0.4) | 0.0137 | −0.76 |
|  | Hind | Symmetry PR | −1.4 (−2.7 to −0.36) | 0.0137 | −0.76 |
|  | Front | Normalized energy | −0.247 (−2.6 to −0.036) | 0.0195 | −0.73 |
|  | Front | Energy | −4.5 (−77 to −0.058) | 0.0371 | −0.66 |
|  | Hind | Stance Phase | +0.0375 (+0.0075 to +0.065) | 0.0371 | +0.66 |
|  | Hind | Step regularity | −1.57 (−3.6 to −0.27) | 0.0488 | −0.63 |
| **Intact female** | Hind | Symmetry BF | −4.01 (−11 to −0.27) | 0.0312 | −0.90 |
| **Intact** | Hind | Swing Phase | −0.065 (−0.095 to −0.017) | 0.0266 | −0.61 |
|  | Front | Maximum acceleration amplitude | +1.05 (+0.04 to +2.8) | 0.0398 | +0.57 |
| **Young** | Front | Average ROM | +2.02 (+0.54 to +3.7) | 0.0312 | +0.9 |
|  | Front | Extension | +1.16 (+0.47 to +2.5) | 0.0312 | +0.9 |
| **Adult** | Hind | Stance Phase | +0.0475 (+0.018 to +0.065) | 0.0064 | +0.53 |
|  | Hind | Flexion | −1.04 (−2.2 to −0.26) | 0.0096 | −0.49 |
|  | Hind | Average ROM | +1.5 (+0.03 to +4) | 0.0491 | +0.38 |
| **Medium** | Hind | Energy | +16 (+1.5 to +31) | 0.0137 | +0.72 |
|  | Hind | Symmetry AR | +1.68 (+0.08 to +4.2) | 0.0244 | +0.67 |
| **Large** | Hind | Maximum gyroscope amplitude | +0.445 (+0.086 to +0.84) | 0.0127 | +0.46 |
|  | Hind | Symmetry PR | −1.05 (−1.8 to −0.28) | 0.0127 | −0.46 |
|  | Hind | Vertical force proxy, IMU-derived | −97.5 (−1.9e+02 to −13) | 0.0179 | −0.44 |
|  | Hind | Swing Phase | −0.04 (−0.07 to −0.0075) | 0.0180 | −0.46 |
|  | Hind | Variance ang. vel. joint axis | +0.374 (+0.05 to +0.73) | 0.0281 | +0.41 |
|  | Hind | Symmetry PL | −0.993 (−1.9 to −0.14) | 0.0298 | −0.41 |
|  | Hind | Variance diff. gyroscope signal | +0.135 (+0.009 to +0.26) | 0.0337 | +0.4 |
|  | Hind | Symmetry BP | −0.95 (−1.7 to −0.045) | 0.0358 | −0.4 |
|  | Front | Energy of acceleration | −3.06 (−7.2 to −0.31) | 0.0402 | −0.39 |
|  | Hind | Stance Phase | +0.0275 (+2.8e−17 to +0.053) | 0.0436 | +0.39 |
|  | Front | Vertical force proxy, IMU-derived | −124 (−2.8e+02 to −3.5) | 0.0451 | −0.38 |
|  | Hind | Energy of acceleration | +5.07 (+0.0073 to +9) | 0.0477 | +0.37 |
